# Psychosocial consequences of receiving false-positive colorectal cancer screening results: a qualitative study

**DOI:** 10.1080/02813432.2019.1608040

**Published:** 2019-05-11

**Authors:** Eva Lykke Toft, Sara Enggaard Kaae, Jessica Malmqvist, John Brodersen

**Affiliations:** a Center for Research & Education in General Practice, Copenhagen, Denmark;; b Primary Health Care Research Unit, Region Zealand, Denmark

**Keywords:** Colorectal cancer, mass screening, colonoscopy, immunochemical faecal occult blood test, qualitative research, psychosocial consequences, harms

## Abstract

**Objective:** The objective of this study was to investigate the psychosocial consequences of receiving false-positive colorectal cancer (CRC) screening results, following a positive immunochemical faecal occult blood test.

**Design, setting, and subjects:** We conducted a qualitative study with four semi-structured focus group interviews with 16 participants aged 50–74, all of whom had received a false-positive result in the national Danish CRC screening programme. We selected, recruited, and grouped participants to ensure maximum variation, and to enable a level of confidence to speak openly about experiences of screening. We subjected interview data, audio-recordings, and transcripts to a strategy of qualitative analysis called systematic text condensation.

**Results:** We identified four main themes which described the psychosocial consequences of false-positive CRC screening results: anxiety; discomfort; changed self-perception and behaviour; and considerations on participation in screening. Each of these themes covered a wide range of experiences which were relevant to the informants and broadly shared by them in many aspects.

**Conclusions:** Receiving false-positive results from CRC screening can lead to negative psychosocial consequences such as changes in self-perception and anxiety: some participants may experience subsequent relief, others not. These negative psychosocial consequences might persist over time.

**Implications:** Negative psychosocial consequences from false-positive CRC screening results may result in a greater use of general practitioner services by healthy people who need reassurance or further tests. More research using condition-specific measures is required to further understand the degree and potential persistence of psychosocial consequences of false-positive results from CRC screening.

**Key Points**
- Participants who receive false-positive colorectal cancer (CRC) screening results may experience negative psychosocial consequences e.g. anxiety and subsequent relief.
- Participants who receive false-positive CRC screening results may experience discomfort during the screening process.
- Participants who receive false-positive CRC screening results may experience longer term changes of self-perception.
- Participants who receive false-positive CRC screening results may experience ambivalence about the offered diagnostic down-stream procedures including colonoscopy.

## Introduction

Mass medical screening is a broadly accepted but fairly blunt instrument that roughly separates screening participants into two groups: one with a high risk and one with a low risk of having the condition screened for. Screening has the potential to lead to intended benefits but also unintended harms [1–3]. The benefits of screening must outweigh the physical, psychological, and social harms to the individual as well as the economic and social consequences for society [1,4].

**Figure 1.**
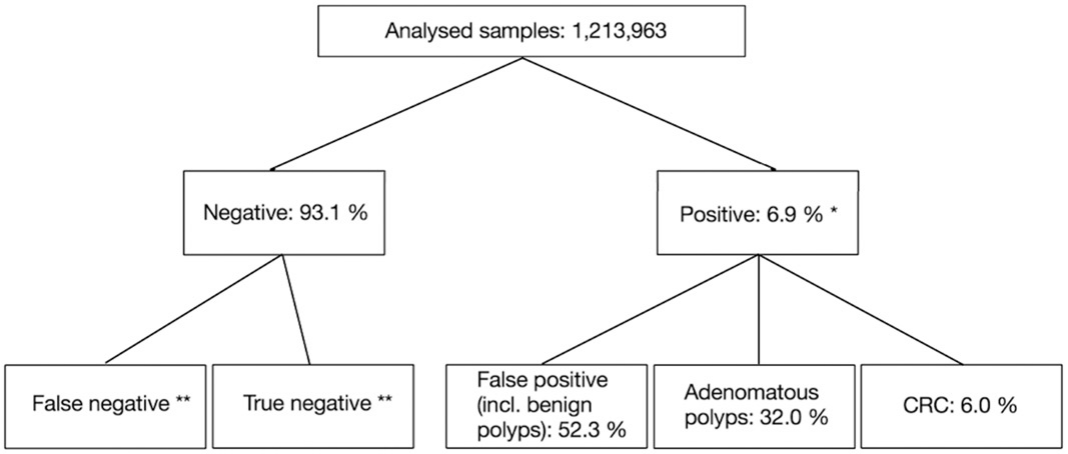
The results from the national Danish CRC prevalence screening round (2014-2017) [6]. Note: *Approximately 10% of these chose not to undergo follow-up colonoscopy; **Not yet known for the full period (2014-2017).

Screening for colorectal cancer (CRC) with faecal occult blood test (FOBT) has shown a relative reduction in disease-specific mortality [5] and has been implemented in several countries. In Denmark, a national CRC screening programme using immunochemical FOBT (iFOBT) as screening method, was implemented 1 March 2014. According to the latest CRC screening report from 2016, of citizens with a positive iFOBT result, 62.9% received a false-positive result (including benign polyps), 31.3% had adenomatous polyps, and 5.8% were diagnosed with CRC [6] (Figure 1).

### Presuppositions

The Danish Medical Education includes education in secondary preventive initiatives such as population screenings. Furthermore, it is suggested that these might result in substantial individual as well as socioeconomic benefits. Danish medical students are also taught that first of all, physicians must do no harm. Therefore, we found it important to also investigate the adverse effects of a CRC screening programme.

JM has research experience in lung cancer and CRC screening. JB has extensive research experience in measuring patient reported outcomes in cancer screening using both qualitative and quantitative methods [7]. JBs previous research has shown that receiving false-positive screening results can lead to negative psychosocial consequences and an increased use of healthcare services [8].

To our knowledge, the international literature on psychosocial consequences from CRC screening mainly consists of quantitative studies using generic questionnaires [9–14]. The results are diverging, and existing evidence [15] suggests, that generic questionnaires are not able to capture all the facets of potential condition-specific psychosocial consequences. Furthermore, only one study measured psychosocial consequences in a Danish setting [13]. We have not been able to identify any qualitative studies that explore this aspect of CRC screening. Therefore, the aim of this qualitative study was to investigate the psychosocial consequences of receiving false-positive results in a CRC screening programme, using iFOBT as the screening method.

## Methods

### Selection, recruitment, and grouping of the informants

We conducted four semi-structured focus group interviews to explore the possible psychosocial consequences of false-positive CRC screening results. The focus group interviews took place in a non-hospital environment at the Head Office of Region Zealand, Denmark.

The CRC Screening Unit in Region Zealand located in Naestved municipality, provided us with a list of 218 people, aged 50 years or older, who had received false-positive results. We conceptualised that a false-positive result was based on an individual receiving an iFOBT result ≥100 ng/mL, who subsequently had a colonoscopy examination, showing a clean colon (no abnormalities) or benign polyps.

The list of potential participants had their positive iFOBT between 1 June 2014 and 31 May 2015. We divided the list into four focus group categories based on gender (M/F), and colonoscopy result (clean colon or benign polyps) and selected 20 potential participants from each of these lists. In addition, the 80 individuals were selected by strategic sampling attempting to achieve maximum variation in age and in date of screening result. In this way of sampling, we attempted to include participants with both possible short and longer-term psychosocial consequences. Participants were also selected by convenience sampling from a geographic area close to the Head Office of Region Zealand, namely the municipalities of Sorø, Slagelse and Ringsted. Each of these municipalities has both urban and rural populations. We arranged the four focus groups as shown in Table 1, Appendix 2.

Previous experience with focus group interviews has shown that of the number invited, approximately one fourth chooses to participate [7]. According to Halkier [16], focus groups discussing personal issues and illness should be relatively small (approximately six participants). Invitations, along with a brief description of the study aim, were sent by mail via the screening unit in Naestved during September and October 2015. We recruited participants parallel to the interviews which were conducted between October and November 2015.

No identifying factors appeared on the lists of screening participants. Thus, the identities of the informants were unknown to us until they replied to the invitation by e-mail, telephone, or text message, consenting to participate.

### Data collection

At the beginning of each interview, we introduced ourselves, our backgrounds, the purpose of the study, and the general purpose of a focus group interview. All interviews had two parts: we conducted the first part using an interview guide, and in the second part we tested a screening-specific questionnaire for use in parallel studies. If new information emerged from the questionnaire, we transcribed it and analysed it with data from part one.

The interviews were semi-structured and we (ET, SK, and JB) took turns to serve as moderators, take notes, and ask elaborating questions. JM participated in the latter three interviews by observing and asking questions. After each interview, we evaluated the information provided by the informants and discussed whether more interviews were needed to reach data saturation. After conducting four focus group interviews, data saturation was achieved as no new information was provided in the latter two group interviews. Between each interview, we conducted initial thematic condensation of the transcripts and adjusted the interview guide if new relevant elements were presented.

The interview guide was built around a chronological examination of the informants’ thoughts, feelings, and experiences during their participation in CRC screening. We asked questions about their experience of: 1) receiving the invitation to participate in the CRC screening programme, 2) receiving the letter with the positive iFOBT result, 3) the waiting period between the positive iFOBT result and the colonoscopy, 4) the process of the bowel preparation, 5) the colonoscopy examination itself, 6) the waiting period until the result of the colonoscopy, and, finally, 7) we asked what they thought of medical screening in general and CRC screening specifically, now that some time had passed. We asked questions about any changes in everyday habits, relationships to family and friends, and self-perception during and after their participation in screening. Questions were primarily open-ended to encourage a dialogue among the informants and to endorse nuanced responses.

All interviews were audio-recorded and lasted between 55 and 90 minutes.

### Data analysis

For the purposes of the analysis, we identified informants according to their colonoscopy result (clean colon [CC], or polyps [P]), gender (M/F) and a random number between 1 and 5 depending on the number of informants who participated in each group. Two authors (ET and SK) transcribed the interviews verbatim and independently coded them.

All audio-recordings and transcripts were subjected to a strategy of qualitative analysis called systematic text condensation [17]. Firstly, the transcripts were each read several times while listening to the audio recordings to get a total impression of the whole material and to ensure that the transcripts stayed true to the recordings. This process started between interviews, and as more interviews were conducted, different preliminary themes came to our attention as recurring across the focus group interviews. Next began a process of manually identifying information-rich sections – meaning units – from informant statements which could possibly elucidate our research question. The relevant text elements were marked in the material by colour codes and subsequently copied out of the transcripts. This was a process of decontextualising the meaning units, taking them from their original context to analyse them alongside meaning units from the other interviews. We strived at not being too restrictive at this point, as we would rather include too much material than too little.

The process of meaning condensation started by two authors (ET and SK) independently categorizing and coding the meaning units into thematic subgroups depending on how and if they fit into the preliminary themes. This coding process was dynamic, as it was occurring alongside the conduction of interviews. Hence, we could continuously evaluate which aspects of each thematic subgroup that were relevant to our research question. The meaning units of each subgroup were then reviewed, and the content was reduced to condensates. These condensates were summaries, constructed by us, which were intended to contain the essence of each subgroup. Quotations (Appendix 1, Quotations in Danish) were chosen to illustrate the condensates and were translated ad hoc with assistance from a native English speaker who has lived in Denmark for more than 20 years. Condensates and their complementary quotations are presented in the result section.

During the process, we discovered that some thematic subgroups needed simple recoding to become more distinct, while others turned out to cover different nuances of an aspect or more than one phenomenon and thus deserved splitting into two or more subgroups. Labelling of preliminary themes was also adjusted continuously as our understanding of the material evolved. Allowing for this flexibility promoted that the labelling continuously represented the most accurate definition and description of the findings.

A complete version of the transcripts, which was neither coded nor decontextualised, was maintained. This was used as a reference point to ensure that our synthesised results still reflected their original context – hereby recontextualising and validating our findings.

During the analysis, disagreements among the authors were resolved by re-auditing the recordings and through discussion until we reached consensus. Finally, all audio-recordings were deleted.

## Results

Of 80 people invited, 17 persons agreed to participate in the four interviews, with 16 informants showing up (characteristics of informants are listed in Table 1, Appendix 2).

During data analysis, we extracted 469 meaning units from the interviews. These were categorised into 19 thematic subgroups, from which we in the end derived four overarching themes: ‘anxiety’, ‘discomfort’, ‘changed self-perception and behaviour’, and ‘considerations on participation in screening’. Each of these four themes is described in detail below.

### Anxiety

Receiving positive iFOBT results made some informants think about the risk of having cancer and dying from it. Some were initially very frightened. One informant described it as follows:

[1]… but I had already thought all kinds of thoughts. I had already died… Several times. (…) Right when I received it [the letter] I thought I was going to faint. – (CCF3)

Not all informants had the same experience. Some of them said they had refused to believe that they were ill since nothing had felt out of the ordinary, therefore, they had not been worried. Others had not been anxious since they had not seen blood in the stool or on the toilet paper. Some informants, particularly those who had not worried much about the positive screening result, said that their relatives had been worried on their behalf. They described seeing the shock on their close relatives’ faces. During the interviews, however, it became evident that even the informants who claimed not to have worried, felt relieved and happy when they received the result of their colonoscopy examination, in the same way as those who had been worried. Some informants shared a bottle of champagne or wine with their families to celebrate their normal colonoscopy result.

During the interviews, anxiety related to the colonoscopy procedure itself was a specific topic. Physical and emotional symptoms of anxiety such as palpitations or difficulty sleeping were mentioned. Others were worried about pain during the procedure.

[2] I must admit that I was more afraid of the examination than I was of being ill. Before the examination you see a nurse, who puts in a needle [intravenous access]; ‘In case it hurts,’ she says. ‘Oh!’ If I wasn’t already afraid… [everybody laughs], I was now. She explained that I could bring my wife into the examination room. But ‘No!’, there was no reason for her to see how scared I was. So yeah… I was… If I wasn’t worried already, I definitely became worried. – (PM4)

This informant had not been worried initially, but the possible need for intravenous analgesics made him think of the colonoscopy as a frightening and serious procedure.

One informant continued to feel anxious more than a year after her colonoscopy examination. Anxiety flared up whenever she noticed any change in her stool or if something else reminded her of the CRC screening. Other informants experienced similar recurring bursts of CRC anxiety related to specific situations or topics.

### Discomfort

Pain and discomfort during the bowel preparation process and in relation to the colonoscopy examination itself were widely discussed during the interviews. Some informants described the colonoscopy as an invasion of private boundaries:

[3] And I also think that it was crossing my personal boundaries. I think so. Definitely. (…) I really think it was unpleasant. I sure didn’t like to be exposed at that end. I felt… But I would do it again. (…) But it was natural to give birth. This here is because you might be ill, right?! That is different I think. – (PF2)

Insufflation of air into the colon during the examination was a painful experience for some informants, but it also made one informant worry about ‘decent behaviour’. She expressed how embarrassing it would be to accidentally fart in the colonoscopist’s face. Another informant worried that the examination would be unpleasant for him ‘as a man’, because the scope was entering through the anal canal.

The unpleasantness and inconvenience of the bowel preparation was also discussed. Feeling bound to one’s home and bathroom was mentioned by some of the informants. They found it unpleasant having to share the toilet with other family members and guests while doing the bowel preparation. Also, the amount of laxative and the artificial orange taste were mentioned as distressing. To some informants, this was far more unpleasant than the colonoscopy examination itself.

### Changed self-perception and behaviour

Some informants described their relatives as being extra helpful and caring during the screening process. Some expressed that CRC screening had strengthened their relationships with relatives or friends who had been through something similar. They empathised with each other’s experiences. At one point, a male informant said:

[4] Nothing is as good for love as illness. – (CCM3)

Others explained that they had only shared their experiences with very few relatives. Some informants even said that they had somewhat isolated themselves from friends and family during the screening process. The informants presented different reasons for this seclusion. Some of them felt that it would be uncomfortable or awkward to be confronted with questions regarding their screening experience or results. Others kept it to themselves because they wanted to protect a loved one from getting anxious.

During the screening process, some informants felt overmedicalised. They felt that the health authorities forced the role of a patient on them even though they felt healthy. One informant felt ill when she drank the laxative; she said it made her feel like a patient, and this had amplified her anxiety.

Some informants had not been completely able to let go of the thought that something might still be wrong, although no CRC was found during their colonoscopy examination. One of the moderators summed up on this discussion between some informants, and one informant answered in the following way:

[5] [Have you changed your self-perception of being a healthy person because you have been called back for a colonoscopy again in two years?] I hadn’t thought of it like that. But I have… Really, yes. They want to keep an eye on what might really be something after all. That has caused some doubt I would say. Because that was the only thing that… Of course I was happy when I saw that I wasn’t ill, but then that [invitation to 2-year follow-up (surveillance) colonoscopy] came. I wasn’t very proud of that. – (PM4)

Being enrolled in a surveillance programme due to polyps had surprised him and made him wonder if, in fact, there could be something wrong after all.

### Considerations on participation in screening

Due to various gastrointestinal symptoms, some of the informants had recently had an iFOBT via their general practitioner (GP) – before being invited to CRC screening. A subsequent colonoscopy examination was performed if the iFOBT was positive. No CRC was found nor suspected after relevant diagnostic work-up. Thus, a general thought among these informants when invited to the CRC screening programme had been not to have screening iFOBT or (once they had received the positive iFOBT result) not to accept the follow-up colonoscopy.

One woman had initially decided not to have the iFOBT, but then she received a reminder:

[6] … where I was told to… You know, that it would be good if I did it. And then I did. – (PF4)

The reminder had given her a feeling of ‘being told to do something’, and indeed, she changed her mind and performed the screening test. Other informants also explained that they had felt obliged to participate in the screening programme. Some explicitly said that they simply did as they were told by a physician or another authority.

Family was also mentioned as an important external influence on informants’ choice to participate in the screening programme. One informant said that he participated for the sake of his family, and that it would not have been acceptable in his family to say no to a screening offer.

Some informants said that they would blame themselves if they later got CRC, had they refused the offer of screening. They had little empathy for people who chose not to go to regular screenings.

Others participated to confirm ‘what they already knew’: that they were ‘healthy’. The thought that their test result could be anything but normal had not crossed their minds.

The following quote describes the ambivalent feelings of an informant receiving his positive iFOBT result:

[7] And then I walk around wondering whether I should accept the colonoscopy or not. When all comes to all, would I rather not know?! I argue back and forth like that. – (CCM3)

Others shared this ambivalence about the diagnostic downstream procedures; during the screening process, they found themselves facing feelings and thoughts they had not anticipated - let alone prepared for. Despite these thoughts, some informants said that they would probably participate in the iFOBT screening and a potential follow-up colonoscopy if they were invited again. They expressed great confidence in the screening programme and felt safe being ‘in the system’. Some accepted the discomfort because they believed it was necessary:

[8] I have to endure it. They are not doing it for the fun of it. – (PF1)

The informants expressed gratitude for the opportunity to detect ‘it’ so early that it would not have evolved. Some assumed that if they were diagnosed with CRC as a result of screening, it would be at an early and treatable stage.

[9] Yeah, but as I see it, the good thing is that if they discover a serious problem you can only hope and expect that it will be noted in time, right? So that there can be recovery, or a good outcome. – (CCF1)

## Discussion

### Principal findings

Some informants experienced negative psychosocial consequences from having false-positive CRC screening results, such as anxiety, discomfort, changed self-perception and behaviour, and considerations on participation in screening.

### Strengths and weaknesses

We aimed to reach maximum variation among our informants based on municipality, age, gender, and time since iFOBT. Seeking variation in time enabled investigation of both short and longer-term psychosocial consequences, which is a key strength.

The retrospective study design could be considered a limitation, i.e. the fact that we asked our informants about their feelings *after* they became aware that their result was a false positive might have influenced their answers. However, the use of focus groups was a strength. We facilitated discussions among informants so that they could help each other recall their feelings and experiences. Another benefit of the focus groups was that they enabled informants to reach a level of comfort and trust with each other, so those who had initially not intended to share their feelings, also felt safe to do so. As some aspects of the discussion in the interviews touched on delicate matters, this was of special importance to the study design.

We performed the analyses of this study on transcripts from the interviews. Thus, nonverbal and subtle communication were not available for analysis, except for points written as field notes during interviews or points that we remembered from being present. Additionally, each interview was followed by a debrief between all authors allowing room for discussion of results and evaluation of the interview guide. Furthermore, all analyses were conducted by more than one author (ET and SK). Had the interviews been recorded on video, conversation analysis could have contributed further to our findings.

The general positive attitude among our informants after receiving false-positive CRC screening results could in part be due to participation bias. Previous research has shown that volunteers in medical trials and participants in screening programmes are in general more psychologically robust and resourceful than those who choose not to participate [18]. Bearing this in mind, it could be hypothesised that some of the people who did not want to participate in the present study might have experienced more negative psychosocial consequences than the informants of this study. Therefore, it is appropriate to speculate whether our results would have been less conservative if the many non-responding individuals had participated. This could raise concern of the likelihood of obtaining actual data saturation. However, it might not be the spectrum of experiences that differ between our informants and the non-participants. The differences might be limited to the degree and the duration of these experiences and the outcomes “degree” and “duration” were not within the scope of this qualitative study. Therefore, data saturation is most likely achieved for the spectrum of psychosocial consequences of receiving false-positive CRC screening results.

The size of the focus groups could be considered another possible limitation. However, in small focus group interviews it is easier to discuss sensitive topics and to make sure that everyone is heard. The risk when conducting few or small focus groups is that the sense of data saturation appear by coincidence. Larger focus groups have the potential advantage of introducing a wider range of perspectives, having more people who remember and remind each other and therefore potentially report more diverse information. On the other hand, in large focus groups there is a risk of grouping among the informants, and some might not feel comfortable enough to report personal experiences [16]. Therefore, we sought relatively small focus groups although one or two more informants in three of the groups would have been preferable.

To our knowledge, all the informants in the present study were of Danish ethnicity although ethnicity exclusion was not part of our study design. Thus, we have not investigated the possible psychosocial consequences in a more ethnically diverse population.

We did not issue invitations to participate in our study directly but rather used the Screening Unit in Naestved. Some people may have chosen not to participate because they thought our project was associated with the screening programme or with the health authorities.

### Discussion of results

During the interviews, it became clear that previous encounters with life-threatening diseases or severe illness had an influence on the psychosocial consequences experienced by the informants during and after the screening process. For example, a man who had been very anxious had recently been treated for prostate cancer. Receiving false-positive results might worsen existing anxiety or psychologically vulnerable conditions.

In the future, more and more screening participants will live with cancer because of two mechanisms: 1) more people are (over)diagnosed with cancer and 2) treatments of some cancers have improved [19]. This could lead to more screening participants experiencing the levels of anxiety that we found in this study.

Bowel preparation and the colonoscopy procedure itself were factors that our informants found particularly distressing. This issue of pre-colonoscopy anxiety has been emphasised in a systematic review [20]. Some of our informants felt that the procedures overstepped their personal boundaries. Similar experiences have been described in the context of a cervical cancer screening programme [21]. Although these feelings of discomfort to some participants are an immediate effect of the screening, it can hardly be expected to have long lasting effects. However, it might be possible to prepare participants better by evaluating the pre-procedure information given to them.

Both during and after CRC screening, some informants said that their self-perception had changed. From thinking of themselves as healthy, they started to feel that they might be ill, and they saw themselves as patients. One had been distressed by the fact that, although he was free of cancer, he would be enrolled in a surveillance programme to keep an eye on him after the removal of a benign polyp. Consistent with these findings of changed self-perception, others have argued that being enrolled in a surveillance programme can make a healthy individual feel like a patient [22]. One could say that the line separating the healthy from the ill is narrowing as more screening programmes are implemented. By definition overdetection and overtreatment result in a number of people unnecessarily receiving a label [23]. Even if the label says that nothing is malignant (right now), some are still enrolled in surveillance programmes.

Many of our informants had to some extent felt it was an obligation to participate in CRC screening. Some of them presented this as a negative aspect of screening. This could be due to the dissatisfaction some informants felt after being aware that they had gone through the screening to find that their result was false positive. We might have found something different, had we spoken to them before they received their final results. Others felt compelled to ‘do as they were told’ when a screening invitation came from a health authority, even though their result turned out to have been a ‘false alarm’. Had the outcome been CRC, then the view on ‘being compelled’ to participate in screening might as well have been more positive in general.

An interesting finding was that some informants expressed scant empathy towards those who choose not to be screened for cancer. It raises the question of whether people who opt out feel stigmatised by friends, family, and society. Especially if they are subsequently diagnosed with the disease they refused to be screened for. Other studies have reported that choosing not to comply with existing screening programmes or guidelines is perceived as ‘abnormal’ or ‘irrational’ [24], and it could result in people who are subsequently diagnosed with cancer blaming themselves for getting ill [25].

Some informants intended to participate in the next CRC screening round when invited, despite the discomfort, uneasiness, and anxiety the latest false-positive result had caused them. This appreciative attitude towards the screening programme could partly be a consequence of the relief they felt when they found out that their final result was false positive. The feeling of relief might as well have been a result of a positive and reassuring screening experience. Other screening participants who received a false-positive result might have had a more negative screening experience and another view on the screening programme, e.g. if they had experienced an adverse or unintended event such as extraordinary post-colonoscopy pain or perforation of the bowel. Even the informants who mentioned that they had not worried about the positive iFOBT result said that they celebrated when they received a ‘normal’ colonoscopy result, indicating that they felt there was something to be relieved about. However, as noted by Gram et al [26] after reporting this feeling of gratitude in women following a false-positive mammogram result: ‘It would be unreasonable to put this impact on the positive side of the balance sheet of breast cancer screening since first the fear, then the relief, are induced by the same screening’. There cannot be relief without preceding anxiety. Therefore, we believe that relief should be considered a negative psychosocial consequence of false-positive screening results.

### Implications for practice and research

Experiencing negative psychosocial consequences from receiving false-positive cancer screening results could lead to more frequent visits to the GP as is the case among participants with false-positive screening results in other screening trials [27–29]. It is open to discussion whether an increased use of GP services by ‘healthy people’, induced by screening, is an acceptable way of spending healthcare resources, or if it is an unintended consequence of screening programmes.

Although we found that some informants experienced psychosocial consequences from receiving false-positive results, the qualitative design of this study must be considered. Our results cannot be generalised nor be assumed to portrait the actual prevalence of these psychosocial consequences in the general population. However, it should create awareness regarding the fact that some people do experience more severe and longer lasting psychosocial consequences after receiving false-positive screening results. Furthermore, it should encourage interest in further research hereof. We encourage GPs to bear our results in mind when offering advice on screening to healthy individuals or when meeting those who have experienced false-positive CRC screening results [30].

More research using condition-specific measures is warranted to further understand the degree and potential persistence of the psychosocial consequences described in this study and clarify possible predictors related to these. These facts could also contribute to improving the pre-procedure information on CRC screening and colonoscopy and thereby maybe reduce anxiety.

## Conclusion

People who participate in CRC screening and receive false-positive results can experience negative psychosocial consequences.

## Appendix Appendix 1 – Quotations in Danish

**Table:**

[1] … men jeg nåede da at taenke alt muligt. Jeg var da allerede død… Et par gange. (…) Lige da jeg fik den, der troede jeg, at jeg skulle besvime; Da jeg laeste brevet. – **CCF3**
[2] Jeg var mere bange for undersøgelsen, end jeg var for, at jeg var syg, det må jeg sgu aerligt erkende (..) Først kommer man ind til en sygepl… hvor man får sat sådan en nål i; ”Fordi hvis nu det gør ondt,” siger hun. ”Nåh!” Var jeg ikke bange i forvejen … (alle griner). Så gjorde den da dér. Hun forklarede så at jeg kunne få min kone med derind, og ”narhj, det var der sgu ingen grund til, at hun skulle se hvor bange jeg var”. Ja, så… Jeg blev sådan… Hvis ikke jeg *var* urolig, så *blev* jeg det i hvert fald. – **PM4**
[3] Og jeg vil også synes, at det var lidt graenseoverskridende. Det synes jeg da også. Helt bestemt. (…) Jeg synes altså, at det var ubehageligt. At ligge blottet i den ende, det brød jeg mig da ikke om. Ja, jeg følte mig… Men jeg ville da gøre det igen. (…) Men det var naturligt at føde et barn. Det andet her, det var jo fordi man måske fejler noget, ikke?! Det er noget andet synes jeg. – **PF2**
[4] Der er ikke noget som sygdom, der er godt for kaerligheden. – **CCM3**
[5] [Har du aendret dit billede af dig selv som rask i forbindelse med, at du nu er blevet indkaldt til sådan en (kikkertundersøgelse) igen om to år?] Sådan havde jeg ikke taenkt på det. Men jeg har… Ja, egentlig. Altså de vil holde øje med at der var måske alligevel et eller andet. Det har sået en tvivl vil jeg sige. Fordi det var det eneste der… Selvfølgelig var jeg glad da jeg så, at jeg ikke fejlede noget, men så kom den der [besked om behov for opfølgende koloskopi]. Den var jeg ikke så stolt af. – **PM4**
[6] … hvor jeg fik at vide, at jeg sku.. Altså, at det ville vaere godt, hvis jeg gjorde det, og det gjorde jeg så. – **PF4**
[7] Og så går jeg og spekulerer lidt på, om jeg skal sige ja til kikkertundersøgelsen, eller skal jeg ikke? Vil jeg I virkeligheden ikke have det at vide, hvis det var?! Sådan går jeg og taenker lidt frem og tilbage. – **CCM3**
[8] Jeg skal holde det ud, fordi de gør det jo ikke for deres morskabs skyld. – **PF1**
[9] Jo, men det gode er, som jeg ser det, at hvis nu man har konstateret en ting som var alvorlig, så må man jo håbe og regne med, at det bliver konstateret i tide eller i god tid, ikke? Så kan der vaere en helbredelse eller noget, hvor det går godt. – **CCF1**

## Appendix Appendix 2.

**Table 1.** Information on informants

	1st focus group interview	2nd focus group interview	3rd focus group interview	4th focus group interview	In total
Gender	Female	Male	Female	Male	
Colonoscopy result	Polyps	Clean colon	Clean colon	Polyps	
Invited	20	20	20	20	80
Attended	5	3	4	4	16
Age	(55–71)	(62–66)	(50–75)	(58–75)	
Coding of informants	PF	CCM	CCF	PM	
